# Glycemic variability: adverse clinical outcomes and how to improve it?

**DOI:** 10.1186/s12933-020-01085-6

**Published:** 2020-07-04

**Authors:** Zheng Zhou, Bao Sun, Shiqiong Huang, Chunsheng Zhu, Meng Bian

**Affiliations:** 1 Department of Chinese Medicine, The First Affiliated Hospital of Zhengzhou University, Zhengzhou, 450000 China; 2 Department of Clinical Pharmacology, Xiangya Hospital, Central South University, Changsha, 410000 China; 3 Hunan Key Laboratory of Pharmacogenetics, Institute of Clinical Pharmacology, Central South University, Changsha, 410000 China; 4 Department of Pharmacy, The First Hospital of Changsha, Changsha, 410005 China

**Keywords:** Glycemic variability, Short-term glycemic variability, Long-term glycemic variability, Adverse clinical outcomes, Beneficial measures

## Abstract

Glycemic variability (GV), defined as an integral component of glucose homoeostasis, is emerging as an important metric to consider when assessing glycemic control in clinical practice. Although it remains yet no consensus, accumulating evidence has suggested that GV, representing either short-term (with-day and between-day variability) or long-term GV, was associated with an increased risk of diabetic macrovascular and microvascular complications, hypoglycemia, mortality rates and other adverse clinical outcomes. In this review, we summarize the adverse clinical outcomes of GV and discuss the beneficial measures, including continuous glucose monitoring, drugs, dietary interventions and exercise training, to improve it, aiming at better addressing the challenging aspect of blood glucose management.

## Background

Glycemic variability (GV), referring to oscillations in blood glucose levels, is usually defined by the measurement of fluctuations of glucose or other related parameters of glucose homoeostasis over a given interval of time (i.e., within a day, between days or longer term). Although HbA1c was traditionally considered as the gold standard for assessing glycemic control [1], GV is a more meaningful measure of glycemic control than HbA1c in clinical practice, and is without doubt now being recognized [2].

**Fig. 1:**
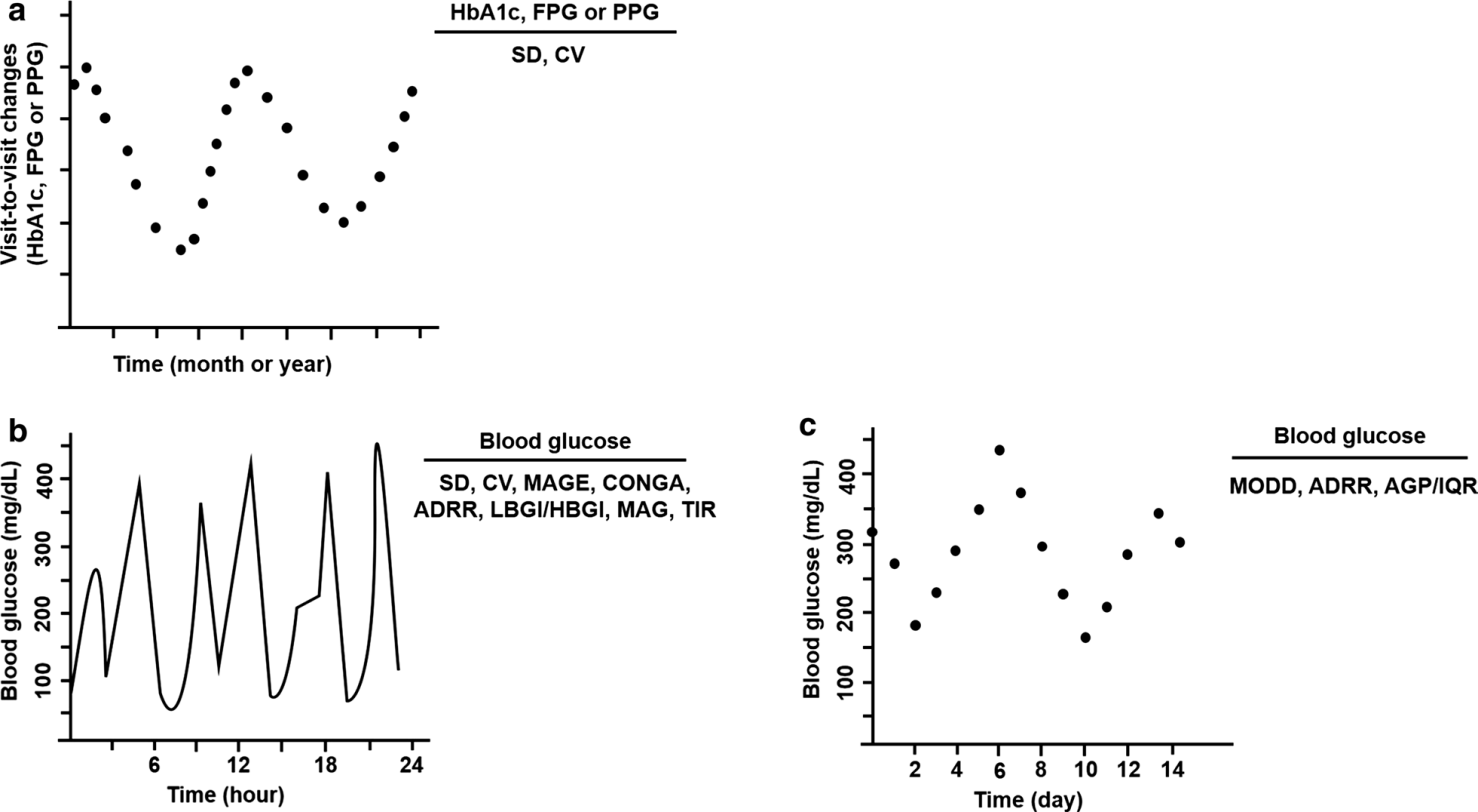
Two principal types of GV. **a** Long-term GV based on visit-to-visit changes of HbA1c, FPG or PPG. **b**, **c** Short-term GV represented by within-day and between-day GV. GV, glycemic variability; FPG, fasting plasma glucose; PPG, postprandial glucose; SD, standard deviation; CV, coefficient of variation; MAGE, mean amplitude of glycemic excursions; CONGA, continuous overlapping net glycemic action; MAG, mean absolute glucose; MODD, mean of daily differences; AGP, average glucose profile; IQR, interquartile ranges; LBGI, low blood glucose index; HBGI, high blood glucose index; ADRR, average daily risk range; TIR, time in range

Despite its clinical significance, there is no consensus on the optimum method for characterizing GV [3]. Over the years, various metrics quantifying GV have been introduced, but many of them are not well understood [4, 5]. Thus, metrics effectively describing GV will be desirable. There are predominantly two types of GV according to the length of time-interval: long-term GV, based on serial determinations over a longer period of time, involving HbA1c, serial fasting plasma glucose (FPG) and postprandial glucose (PPG) measurements, and short-term GV, represented by both within-day and between-day GV (Table 1). Long-term GV, usually based on visit-to-visit measurements of HbA1c, FPG or PPG [6], with the subsequent calculation of their standard deviation (SD) and coefficient of variation (CV), reflects the surrounding hyperglycemia to a certain extent, because measures of long-term variability correlate with either mean concentration of blood glucose or mean HbA1c [7, 8] (Fig. 1a). For another type of GV, short-term GV is characterized by sudden and rapid upward or downward glucose changes within- or between-days (Fig. 1b, c). Furthermore, short-term GV is calculated from self-monitoring of blood glucose (SMBG) measurements for a long time [7], but this method has been gradually replaced by continuous glucose monitoring (CGM) over the past few years [9–12]. CGM, with interstitial glucose measurements at 5 min intervals, provides a more comprehensive record during the day and night periods compared to SMBG [7, 10]. Similar to long-term GV, the common metrics of short-term GV include the SD and CV. When averaging each daily SD or CV, the mean of within-day daily GV over the stated time period can also be estimated [4]. Service et al. introduced that the mean amplitude of glycemic excursions (MAGE) was the “gold standard” for assessing the short-term with-day GV [13]. Due to its simplicity, MAGE remained still commonly used to assess the with-day GV by further measuring the arithmetic mean of the differences between consecutive peaks and nadirs. Moreover, a novel approach to measurement of with-day GV was presented by the continuous overlapping net glycemic action (CONGA) metric that calculates the SD of difference between a current blood glucose reading and a reading taken hours earlier [14]. Another metric of with-day GV was the mean absolute glucose (MAG), which summed absolute differences between sequential readings divided by the time between the first and last blood glucose measurement [2]. Correspondingly, the metrics for estimating the between-day GV were referenced to as the mean of daily differences (MODD) and introduced by Molnar et al. [15]. This index assessed the between-day GV based on the calculation of the absolute differences between two glucose values measured at the same time with a 24 h interval. Average glucose profile (AGP), a measure of the between-day GV over a 14-day period, was determined by using the flash glucose monitoring system and reported the results as interquartile ranges (IQRs) [16, 17]. Apart from the above indices, particular attention should be given to the low blood glucose index (LBGI), high blood glucose index (HBGI) and average daily risk range (ADRR), as they were associated with the risk of hypo- and hyperglycemia. Among these indices, LBGI and HBGI were preceded by a log transform to render symmetric the skewed distribution of glucose values [4, 18], and ADRR was sum of the daily peak risks for hypo- and hyperglycemia [19]. Recently, time in range (TIR) was identified as a key metric of glycemic control, and referred to the percentage of time per day within target glucose range (3.9–10.0 mmol/L) [20, 21].

**Table 1:** Main types of metric for assessment of GV

Types of metric	Computation or description	References
Long-term GV		
Visit-to-visit measurements of HbA1c, FPG or PPG	Measures of SD or CV of HbA1c, FPG and PPG between sequential visits	[6]
Short-term GV		
SD	Variation around the mean blood glucose	[4]
CV	Magnitude of variability relative to mean blood glucose	[4]
MAGE	Mean differences from peaks to nadirs	[13]
CONGA	Difference between a current blood glucose reading and a reading taken hours earlier	[14]
MAG	Absolute differences between sequential readings divided by the time between the first and last blood glucose measurement	[2]
MODD	Absolute differences between two glucose values measured at the same time with a 24 h interval	[15]
AGP/IQR	Distribution of glucose data at a given timepoint and resulted as interquartile ranges	[16, 17]
LBGI/HBGI	Preceded by a log transform to render symmetric the skewed distribution of glucose values	[4, 18]
ADRR	Sum of the daily peak risks for hypoglycemia and hyperglycemia	[19]
TIR	Percentage of time per day within target glucose range (3.9–10.0 mmol/L)	[20, 21]

GV, glycemic variability; FPG, fasting plasma glucose; PPG, postprandial glucose; SD, standard deviation; CV, coefficient of variation; MAGE, mean amplitude of glycemic excursions; CONGA, continuous overlapping net glycemic action; MAG, mean absolute glucose; MODD, mean of daily differences; AGP, average glucose profile; IQR, interquartile ranges; LBGI, low blood glucose index; HBGI, high blood glucose index; ADRR, average daily risk range; TIR, time in range

In our previous study, we indicated that GV was correlated with cardiovascular events and hypoglycemia [22]. Although it remains yet controversial, emerging evidence has suggested that GV was associated with an increased risk of microvascular and macrovascular complications, hypoglycemia and mortality rates [23–25]. The aim of this study is to summarize the adverse clinical outcomes of GV and discuss the potential beneficial measures including CGM, drugs, diets and exercise to improve it, aiming to address the challenging aspect of blood glucose management.

## GV and adverse clinical outcomes

Given that the limitations of HbA1c measurements, growing evidence demonstrated that GV was a significant and clinically meaningful glycemic metric and had drawn attention for its effects on adverse clinical outcomes, including diabetic macrovascular and microvascular complications, hypoglycemia and mortality [26–29] (Table 2).

**Table 2:** The effects of GV on adverse clinical outcomes

Types of GV	Subjects	Effects	References
Short-term GV			
TIR	3262 patients with type 2 diabetes	Inversely correlated with DR	[27]
Day-to-day FPG variability	7637 patients with type 2 diabetes	Increased risks of severe hypoglycemia and all-cause mortality	[29]
MAGE	417 patients with ACS	Predicted the poor prognosis for patients with acute coronary syndrome	[32]
Mean daily δ blood glucose	160 patients with transcatheter aortic valve implantation	Increased the risk of macrovascular complications	[35]
MAGE	204 patients with type 2 diabetes	Increased coronary artery disease severity	[36]
MAGE	50 patients with dysglycemia	Positively correlated with coronary artery spasm	[37]
MAGE	2666 hospitalized patients with CAD	Positively associated with poor prognosis in CAD patients	[38]
Incremental glucose peak	2758 patients with type 2 diabetes	Positively associated with aortic stiffness and maladaptive carotid remodeling	[39]
MAGE	40 patients with type 1 or type 2 diabetes	Positively associated with DPN	[51]
LBGI and HBGI	140 patients with type 2 diabetes	Increased the risk of hypoglycemia	[66]
LBGI	73 patients with type 1 diabetes	Increased the risk of hypoglycemia	[67]
Day‐to‐day fasting SMBG variability	1221 patients with type 1 or type 2 diabetes	Increased the risk of overall symptomatic, nocturnal symptomatic and severe hypoglycemia	[68]
CONGA, MAG and MAGE	83 patients with type 2 diabetes	Predicted the nocturnal hypoglycemia	[69]
Mean blood glucose	62 patients with type 2 diabetes	Predicted the hypoglycemia	[70]
CV within a day	6101 critically ill adults	Increased the risk of mortality and hypoglycemia	[72]
IQR	28,353 patients with type 2 diabetes	Increased the risk of mortality	[73]
Long-term GV			
Visit-to-visit variability of FPG	654 patients with type 2 diabetes	Predicted the renal composite outcome	[31]
SD during initial hospitalization	327 patients with diabetes and ACS	Predicted the midterm macrovascular complications	[40]
Visit-to-visit variability of FPG	53,607 patients initially without CVD	Increased the risk of CVD and all-cause mortality	[41]
Visit-to-visit variability of FPG	1791 patients with type 2 diabetes	Positively associated with the risk of CVD	[42]
Visit-to-visit variability of FPG	455 patients with type 2 diabetes	Independently associated with annualized changes in left cardiac structure and function	[43]
Visit-to-visit variability of FPG	3769 patients initially without CVD	Increased the risk of incident diabetes, CVD and mortality	[44]
Visit-to-visit variability of FPG	3,211,319 patients without diabetes	Independently associated with CVD and mortality	[45]
Visit-to-visit variability of HbA1c	632 patients with type 2 diabetes	Predicted the additive risk for CVD incidence	[46]
Visit-to-visit variability of HbA1c	972 patients with type 2 diabetes	Positively associated with macrovascular complication	[47]
Visit-to-visit variability of HbA1c	201 patients with type 2 diabetes	Potentially predicted the progression of HFpEF	[48]
Visit-to-visit variability of HbA1c	902 patients with type 2 diabetes and heart failure	Predicted all-cause mortality	[49]
Visit-to-visit variability of FPG	2773 patients with type 2 diabetes	Positively correlated with DPN	[52]
Visit-to-visit variability of FPG	36,152 patients with type 2 diabetes	Predicted the risk of DPN	[53]
Visit-to-visit variability of HbA1c	563 patients with type 2 diabetes	Positively associated the risk of DPN	[54]
Visit-to-visit variability of HbA1c	220 patients with type 1 diabetes	Positively associated the risk of DPN	[55]
Visit-to-visit variability of HbA1c	223 patients with type 2 diabetes	Positively associated with the severity of DPN	[56]
Visit-to-visit variability of HbA1c	451 patients with type 1 diabetes	Increased the risk of DR	[58]
Visit-to-visit variability of HbA1c	895 patients with type 2 diabetes	Positively associated with progression of DN	[60]
Visit-to-visit variability of HbA1c	4231 patients with type 2 diabetes	Increased the risk of DKD	[61]
Visit-to-visit variability of HbA1c	1383 patients with type 2 diabetes	Increased the deterioration of renal function	[62]
Visit-to-visit variability of HbA1c	388 patients with type 2 diabetes	Positively associated with renal progression	[64]
Visit-to-visit variability of FPG	3569 patients with type 2 diabetes	Increased the risk of mortality	[74]
Visit-to-visit variability of HbA1c	15,733 patients with type 2 diabetes	Strongly predicted all-cause mortality	[75]
Visit-to-visit variability of FPG	1136 patients with type 2 diabetes	Predicted all-cause mortality	[76]
Visit-to-visit variability of FPG	42,418 hypertensive patients	Increased the risk of mortality	[77]
CV and SD during hospitalization	20,303 hospitalized patients	Increased longer hospitalization and mortality	[78]
Visit-to-visit variability of HbA1c	6048 patients with type 1 diabetes	Increased mortality and earlier hospital admission	[79]
Visit-to-visit variability of HbA1c	58,832 patients with type 2 diabetes	Positively associated with overall mortality and emergency hospitalization	[80]
Visit-to-visit variability of HbA1c	9483 patients with type 2 diabetes	Predicted all-cause mortality	[81]
Visit-to-visit variability of HbA1c	837 patients with type 2 diabetes	Predicted depressive symptoms	[83]
Visit-to-visit variability of FPG	3307 adults before the onset of diabetes	Increased the risk of cognitive function	[84]
Visit-to-visit variability of HbA1c	2640 patients with type 1 or type 2 diabetes	Increased the potential risk of later tumorigenesis	[86]

GV, glycemic variability; TIR, time in range; DR, diabetic retinopathy; FPG, fasting plasma glucose; MAGE, mean amplitude of glycemic excursions; ACS, acute coronary syndrome; CAD, coronary artery disease; LBGI, low blood glucose index; HBGI, high blood glucose index; SMBG, self‐monitored blood glucose; CONGA, continuous overlapping net glycemic action; MAG, mean absolute glucose; CV, coefficient of variation; IQR, interquartile ranges; CVD, cardiovascular disease; HFpEF, heart failure with preserved ejection fraction; DPN, diabetes peripheral neuropathy; DR, diabetic retinopathy; DN, diabetic nephropathy; DKD, diabetic kidney disease; SD, standard deviation

### GV and diabetic macrovascular and microvascular complications

There is considerable evidence to support the negative role of GV in the development of diabetic macrovascular and microvascular complications [22, 30–33].

#### GV and diabetic macrovascular complications

An observational trial indicated that GV assessed by the MAGE was an independent predictive factor of poor prognosis for patients with acute coronary syndrome [32]. Moreover, a meta-analysis conducted by Liang et al. reported that high amplitude of GV played a causal role in cardiovascular disease (CVD), and minimizing GV could improve insulin resistance and reduce carotid intima-media thickness, as well as lower the risk of CVD [34]. Similarly, a post hoc cohort analysis showed that GV evaluated by mean daily δ blood glucose was associated with an increased risk of macrovascular complications (e.g., death, stroke and myocardial infarction) after transcatheter aortic valve implantation [35]. In acute myocardial infraction patients with poorly controlled type 2 diabetes, GV represented by MAGE was associated with coronary artery disease (CAD) severity, and suggested that early evaluation of GV might serve as a therapeutic target [36]. Particularly, intraday GV was thought to be associated with coronary artery spasm in patients with dysglycemia [37]. Recently, Pu et al. showed that increased GV on admission might be associated with poor prognosis in CAD patients [38]. Of note, a current study indicated that daily glucose variability represented by incremental glucose peak during an oral glucose tolerance test was independently associated with aortic stiffness and maladaptive carotid remodeling, but not with microvascular function [39].

In addition to the short-term GV, long-term GV was also strongly associated with the macrovascular complications. Gerbaud et al. reported that GV (cutoff value > 2.70 mmol/L) assessed by SD during initial hospitalization was the strongest independent predictive factor for midterm macrovascular complications in patients with diabetes and acute coronary syndrome [40]. A prospective cohort study including 53,607 Chinese participants found that long-term visit-to-visit variability of FPG increased the risk of CVD (included myocardial infarction, cerebral infarction, and cerebral hemorrhage) and all-cause mortality [41]. In the Veteran Affairs Diabetes Trial (VADT), the adverse consequences of FPG variability on CVD, mainly including myocardial infarction, stroke and cardiovascular death, appeared greatest in patients receiving intensive glucose control [42]. Even more important, visit-to-visit variability in FPG could be a novel risk factor for the long-term adverse changes in left cardiac structure and systolic function [43]. Currently, Bancks et al. suggested that higher intra-individual FPG variability during young adulthood before the onset of diabetes was associated with incident diabetes, macrovascular events and mortality [44]. Noteworthily, Yu et al. even found that long-term FPG variability was independently associated with myocardial infarction and stroke in a general population without diabetes [45]. In addition to the variability of FPG, long-term variability of HbA1c was also correlated with the risk of macrovascular complications. A previous study investigated the association of long-term variability of HbA1c and systolic blood pressure with the incidence of macrovascular complications in patients with type 2 diabetes, and found that they represented a combined and additive risk for macrovascular complications [46]. Moreover, a study identified that long-term variability of HbA1c was associated with macrovascular complication in Chinese type 2 diabetes [47]. Meaningfully, HbA1c variability may provide additional valuable information as a potential predictor for the progression of heart failure with preserved ejection fraction (HFpEF) [48], and was independently and similarly predictive of death or HFpEF [49]. Moreover, GV evaluated by SD of blood glucose level appeared to be an important risk factor for left ventricular diastolic function, and reducing GV may provide a potential new therapeutic strategy for the prevention of the development of HFpEF in T2DM patients [50].

#### GV and diabetic microvascular complications

Likewise, GV played an important role in diabetic microvascular complications. In the Rio De Janeiro Type 2 Diabetes Cohort Study, 24-month visit-to-visit FPG variability was a significant risk predictor for renal outcomes, and 24-month visit-to-visit HbA1c variability was a better risk predictor for diabetic retinopathy progression than HbA1c levels [31]. Akaza et al. revealed that GV estimated by MAGE might be an independently risk factor for diabetes peripheral neuropathy (DPN) in patients with type 1 or type 2 diabetes by using CGM [51]. Specially, long-term FPG variability as represented by the CV was related to the risk of DPN in patients with type 2 diabetes [52]. More importantly, in the National Diabetes Care Management Program, the long-term variability of FPG was considered as one of the potent predictors of DPN in type 2 diabetic patients [53]. On the other hand, researchers disclosed that long-term variability of HbA1c assessed by CV was closely associated with DPN, and was identified as an indicator for DPN in type 1 or type 2 diabetes [54, 55]. Lai et al. performed a cross-sectional study enrolled 223 patients with type 2 diabetes and demonstrated that 3-year visit-to-visit HbA1c variability combined with chronic glycemic impairment was strongly associated with the severity of DPN [56]. They also confirmed that HbA1c variability was independently associated with the severity of cardiovascular autonomic neuropathy [57]. Intriguingly, a recent study showed that GV involved in long-term visit-to-visit HbA1c variability was independently associated with the risk of diabetic retinopathy (DR) in type 1 diabetes [58]. Consistently, Lu et al. revealed that GV estimated by TIR was also strongly associated with DR in patients with type 2 diabetes [27]. Furthermore, a systematic review and meta-analysis ascertained that high FPG variability levels were positively associated with the risk of DR and all-cause mortality in patients with type 2 diabetes [59]. Apart from the DR, GV represented by long-term variability of HbA1c was also significantly associated with the progression of diabetic nephropathy (DN) in type 2 diabetes [60]. The long-term variability of HbA1c, lipid parameters, uric acid and blood pressure influenced the development of DN and had a different impact on albuminuria development and the decline in glomerular filtration rate [61, 62]. Subsequent research clarified that the long-term intra-individual variability in these parameters played a greater role in the progression of DN than the absolute value of each single variable [63]. Importantly, Lee et al. demonstrated that greater HbA1c variability and a decreasing trend of HbA1c was associated with a lower risk of diabetic patients with stages 3–4 chronic kidney disease and poor glycemic control [64]. These findings collectively displayed the pivotal role of GV in diabetic macrovascular and microvascular complications.

### GV and hypoglycemia

Hypoglycemia is the major impediment to therapy in diabetes. While HbA1c remains widely used as a measure of mean glycemia, it may not be the best marker for predicting hypoglycemia. The consolidated evidence to date supported the importance of GV with respect to predicted risk of hypoglycemia [65–67]. Zinman et al. concluded that higher day-to-day FPG variability was associated with increased risks of severe hypoglycemia and all-cause mortality [29]. Moreover, day-to-day fasting SMBG variability was also found to be associated with the risk of overall symptomatic, nocturnal symptomatic and severe hypoglycemia in insulin-treated patients with diabetes [68]. Similarly, the analysis of CGM-derived GV could improve prediction of nocturnal hypoglycemia in elderly patients treated with insulin, and minimizing GV could achieve good glycemic control without hypoglycemia [69, 70]. Additionally, using nested case–control design in electronic health record data in England, Zhong et al. showed that HbA1C variability is a strong predictor for hypoglycemia requiring hospitalization in diabetes [71]. Overall, GV variability may be an important target for hypoglycemia prevention and management in diabetic patients treated with insulin.

### GV and mortality

A number of studies verified that GV was not only associated with the risk of diabetes-related complications and hypoglycemia, but also simultaneously related to the high incidence of mortality [41, 44, 57]. Interestingly, several studies proposed an independent association of GV with mortality [72–75]. Clinical data indicated that FPG variability might be an important predictor of mortality, particularly for those with their glycemic status uncontrolled [76, 77]. Besides, in hospitalized patients, increased GV was associated with a higher rate of mortality [78–80]. Recently, in the Action to Control Cardiovascular Risk in Diabetes (ACCORD) trial, researchers found that HbA1c variability was a strong predictor of all-cause mortality [81], and this observation was more remarkable in older people with diabetes [82].

In addition to the above adverse clinical outcomes, GV was also reported to be associated with depressive symptoms, cognitive disorder and even cancer [83–86]. In the Israel Diabetes and Cognitive Decline (IDCD) study, GV measured as the SD of HbA1c increased the risk of depressive symptoms [83]. A Taiwan diabetes study explored the relationship between GV and the incidence of Alzheimer disease (AD) in patients with type 2 diabetes mellitus, finding that GV had a worse impact on AD and might be significant predictors for AD [84]. More importantly, recent study demonstrated that HbA1c variability was a potential risk factor for later tumorigenesis in patients with diabetes, which might be mediated by oxidative stress or hormone variability [86].

## Potential beneficial measures

There is now cogent evidence for the deleterious effects of GV. As a consequence, it is strongly suggested that potential beneficial measures should be aimed at reducing to a minimum GV (Table 3).

**Table 3:** Potential beneficial measures for addressing GV

Subjects	Measures	Results	References
Patients with type 1 diabetes	CGM	Reduced GV and improved protection against hypoglycemia	[87–89]
	Insulin analogues degludec	Minimized morning GV	[91]
	Canagliflozin	Improved indices of GV	[92]
	Dapagliflozin over 24 weeks	Improved GV without increasing the time spent in the range indicating hypoglycemia	[93]
	Empagliflozin as adjunct to insulin	Decreased glucose exposure and variability and increased time in glucose target range	[103]
	Combination of basal insulin with ipragliflozin or dapagliflozin	Improved TIR and the mean glucose level	[104]
	Low carbohydrate diet	Resulted in more time in euglycemia, less time in hypoglycemia	[108–110]
Patients with type 2 diabetes	Dapagliflozin on 24-h	Improved measures of GV	[94]
	Once-weekly trelagliptin and once-daily alogliptin	Improved glycemic control and reduced GV without inducing hypoglycemia	[95]
	Combination of basal insulin with a GLP-1 RA	Lowered GV and hypoglycemia	[96]
	Exenatide once weekly	Improved daily glucose control and reduced GV	[97]
	Lixisenatide added to basal insulin	Reduced GV and PPG excursions without increasing the risk of hypoglycemia	[98]
	Liraglutide	Lower mean time in hyperglycemia	[99]
	Combination of metformin and gemigliptin or sitagliptin	Significantly reduced GV	[100]
	Vildagliptin or pioglitazone	Significantly reduced MAGE, glycated hemoglobin and mean plasma glucose levels	[101]
	Combination of metformin and vildagliptin or glimepiride	Improved glucose level with a significantly greater reduction in GV and hypoglycemia	[102]
	Intensive insulin therapy combined with metformin	Reduced both glucose fluctuation and nocturnal hypoglycemic risk	[105]
	Low-carbohydrate high-fat diet	Reduced glycemic fluctuation	[106, 107, 111]
	Sequence of food ingestion	Associated with lower post-lunch glucose excursions and lower glucose coefficients of variation	[115]
	Aerobic and combined exercise sessions	Reduced glucose levels and GV	[116–118]
	Short-term exercise training	Improved glycemic control and GV but unaffected oxidative stress	[119, 121]
	Frequent interruptions of prolonged sitting	Improved fasting glucose and night-time glycemic variability	[120]
Others	Low glycemic index foods	Reduced the glycemic response and variability and promoted fat oxidation.	[112, 113]
	Food order	Reduced glycemic excursions	[114]
	Exercise in the fasted and postprandial state	Exercise in the postprandial state after breakfast, but not in the fasted state, decreased glucose excursions	[122]
	Aerobic and eccentric exercise	Reduced all the indices of GV	[123]
	Immediate post-breakfast physical activity	Improved mean, CV and AUC glucose	[124]

GV, glycemic variability; CGM, continuous glucose monitoring; CV, coefficient of variation; GLP-1 RA, glucagon-like peptide 1 receptor agonist; PPG, postprandial glucose; MAGE, mean amplitude of glycemic excursions; TIR, time in range; AUC, area under the curve

### Drugs combined with CGM

Extensive evidence addresses that real-time CGM (rtCGM) improves glycemic control and minimizes the risk of glucose extremes, as well as severe hypoglycemia [87–90]. rtCGM combined with drugs allows a comprehensive analysis of GV and makes timely adjustments. Treatment with insulin analogues degludec, in the context of GV measured by CGM, was related to the lower day-to-day variation in glucose level [91]. In randomized, double-blind studies, canagliflozin and dapagliflozin improved GV in the participants who underwent CGM [92–94]. Furthermore, another randomized pilot study indicated that once-weekly trelagliptin and once-daily alogliptin improved glycemic control and reduced GV without inducing hypoglycemia [95]. Nowadays, greater efficacy is shown in therapies combining new hypoglycemic drugs with insulin or metformin, with improvement in GV also demonstrated by CGM. Bajaj et al. revealed that the combination of glucagon-like peptide 1 receptor agonist (GLP-1 RA) with basal insulin observed the lowest GV and hypoglycemia in type 2 diabetes [96]. In metformin-treated patients with type 2 diabetes, exenatide once weekly significantly improved daily glucose control and reduced GV [97]. Analogously, compared with placebo, lixisenatide added to basal insulin significantly reduced GV and PPG excursions without increasing the risk of hypoglycemia [98]. Furthermore, for type 2 diabetes patients initially treated with insulin, introducing liraglutide had a beneficial effect on GV estimated by CGM [99]. Another new hypoglycemic drugs, dipeptidyl-peptidase 4 (DPP4) inhibitors, combined with metformin therapy improved glucose level with a significantly greater reduction in GV and hypoglycemia [100–102]. A multicenter study compared the GV between DPP4 inhibitor and glimepiride groups, and found that DPP4 inhibitors were more effective than glimepiride in reducing GV as initial combination therapy with metformin in patients with type 2 diabetes [100]. Moreover, other studies demonstrated that vildagliptin reduced GV in individuals with type 2 diabetes ongoing metformin therapy [101, 102]. Consistent results were obtained when combined sodium glucose cotransporter 2 (SGLT2) inhibitors with insulin therapy [103, 104]. Famulla et al. addressed that empagliflozin as adjunct to insulin decreased glucose exposure and variability and increased time in glucose target range in patients with type 1 diabetes [103]. A recent retrospective study unraveled that SGLT2 inhibitors improved TIR without increasing hypoglycemia in Japanese patients with type 1 diabetes [104]. Notably, an observational study indicated that metformin added to initial continuous subcutaneous insulin infusion or multiple daily injections decreased glucose fluctuation and nocturnal hypoglycemic risk in patients with type 2 diabetes [105]. These results clarified that new antidiabetic drugs combined with CGM might be the preferred choice for the reduction of GV.

### Dietary interventions

As an important component of diabetes management, the impact of dietary manipulation on glycemic control cannot be understated. A previous study by Mori et al. demonstrated that the low-carbohydrate/high-monounsaturated fatty acid liquid diet narrowed the range of GV, and might be useful in long-term glycemic control [106]. The current study also compared the effect of glycemic response to low-carbohydrate high-fat diet and high-carbohydrate low-fat diet by using CGM, finding the consistent results [107]. Furthermore, low carbohydrate diet contributed to more time in euglycemia, less GV than high carbohydrate diet [108–110]. Particularly, a very-low-carbohydrate high-fat breakfast appeared to be sufficient to reduce postprandial hyperglycemia and improve glucose excursions [111]. Low glycemic index foods can minimize blood glucose fluctuations and have been advocated to use in diabetic patients. Henry et al. indicated that lower glycemic index foods were able to acutely reduce the GV and promote fat oxidation [112, 113]. Of note, a recent study suggested that the food order (protein or vegetables first, followed by carbohydrate) decreased GV in prediabetes, which presented a novel, simple behavioral strategy to reduce glycemic excursions [114, 115]. In short, effective dietary interventions have the potential to achieve a favorable blood glucose profile by influencing the GV.

### Exercise training

Exercise training, consisting of resistance exercise, aerobic exercise, or a combination of both, is recognized as a frontline therapy for the prevention and treatment of type 2 diabetes. Additionally, previous studies showed that exercise reduced GV or oxidative stress [116, 117], opening a new venue of benefits to explore. There is evidence that different types of exercise have various effects on glucose control. Schein et al. performed a randomized clinical trial and found that inspiratory muscle training decreased glucose levels and GV in patients with type 2 diabetes, which could be a novel exercise modality [118]. Another crossover trial showed that short-term interval walking training improved CGM-derived GV compared with continuous walking training in individuals with type 2 diabetes [119]. Lately, Paining et al. explored that frequent interruptions of prolonged sitting with 3 min of light-intensity walking breaks every 15 min improved night-time GV, which might be an effective approach to improve glucose control [120]. Furthermore, 2 weeks of both high-intensity interval training or moderate-intensity continuous training were similarly effective in lowering GV and endothelial damage [121]. Intriguingly, a randomized study was to test whether moderate exercise performed in either the fasted or the postprandial state affected GV, and concluded that performing moderate exercise in the postprandial state after breakfast tended to decrease glucose excursions compared to the exercise in the fasted state [122].

Recently, in addition to the effect of exercise on GV in patients with diabetes, the same phenomenon was also observed in healthy people. Figueira et al. observed that both aerobic and eccentric exercise reduced GV in healthy individuals, which might be mediated by inflammatory cytokines [123]. Moreover, consistent with the results in patients with diabetes, low- to moderate-intensity exercise soon after breakfast improved GV in healthy people, which will help optimize exercise-meal timing in general health guidelines [124].

## Conclusion and future perspective

**Fig. 2:**
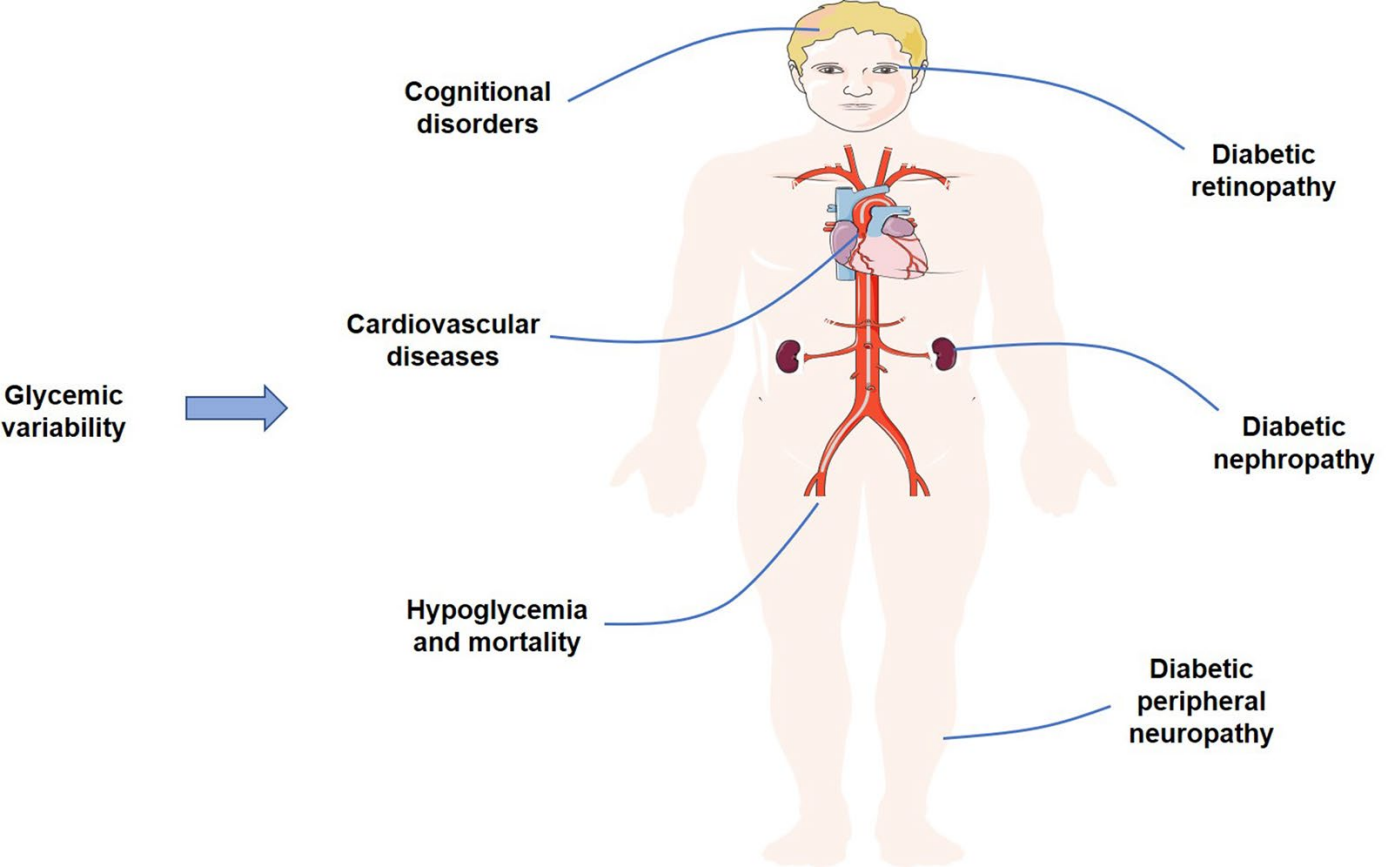
The effects of glycemic variability on the adverse clinical outcomes

We have attempted to summarize the relationships between two categories of GV and the risk for diabetic macrovascular and microvascular complications, hypoglycemia, mortality and other adverse clinical outcomes (Fig. 2). We also generalized the potential beneficial measures including drugs combined with CGM, dietary interventions and exercise training, to improve GV. These findings highlight the important role of GV in the patients with diabetes and provide the essential help for clinicians to manage the blood glucose.

GV has been identified to be closely associated with the risk of adverse clinical outcomes and provides a better predictor of such complications. However, it still lacking a clear universal definition and different indices have been proposed to evaluate it. With the availability of CGM in clinical practice, the assessment of GV became not only possible but also required [2]. Also, CGM was frequently superior to continuous subcutaneous insulin infusion and could guide individuals’ therapeutic changes to reduce GV, hypoglycemia and CVD [125, 126]. A recent study reported that flash glucose monitoring, a new approach to glucose monitoring, has a long sensor lifetime of 14 days and emerged as a practical solution to the glucose monitoring [127]. Meanwhile, a real-world data from Spain indicated that flash glucose monitoring allowed frequent glucose checks and reduced GV, as well as hypoglycemia [128]. Consequently, in order to provide a more comprehensive assessment of GV, the new approach of glucose monitoring is advocated to adopt in clinical practice. Future developments in new technologies, such as CGM systems and flash glucose monitoring, and indices for better deciphering and defining GV should contribute to improve understanding of the clinical relevance of GV in the management of diabetes.

Although GV had drawn attention for its effects on diabetic macrovascular and microvascular complications, hypoglycemia and mortality, several studies have shown conflicting results [7, 129]. Caprnda et al. failed to show the association between diabetic complication and GV in patients with type 2 diabetes [129]. Furthermore, in the Diabetes Control and Complications Trial, within-day GV, as determined from quarterly glucose profiles, did not play an explicit role in the development of microvascular complications [7]. However, we found that these results employed the 7-point glucose profiles, which might be insufficient to characterize GV correctly when compared with CGM. Thus, these negative results may not necessarily disprove the importance of GV in the development of diabetic complications. Additionally, the mechanisms linking GV and related complications risk remained unclear. Recent studies corroborated that GV was correlated with oxidative stress or erythrocyte membrane stability, emphasizing its participation in the pathogenesis of related complications [130, 131]. Further prospective research to explore the explicit mechanisms linking GV and related complications is warranted.

Finally, setting clear definitions and taking potential beneficial measures for addressing GV is essential. Further research in these domains will contribute to blood glucose control and management.

## Data Availability

Not applicable.

## Abbreviations

GV: glycemic variability
FPG: fasting plasma glucose
PPG: postprandial glucose
SD: standard deviation
CV: coefficient of variation
SMBG: self-monitoring of blood glucose
CGM: continuous glucose monitoring
MAGE: mean amplitude of glycemic excursions
CONGA: continuous overlapping net glycemic action
MAG: mean absolute glucose
MODD: mean of daily differences
AGP: average glucose profile
IQRs: interquartile ranges
LBGI: low blood glucose index
HBGI: high blood glucose index
ADRR: average daily risk range
TIR: time in range
CVD: cardiovascular disease
CAD: coronary artery disease
VADT: Veteran Affairs Diabetes Trial
HFpEF: heart failure with preserved ejection fraction
DPN: diabetes peripheral neuropathy
DR: diabetic retinopathy
DN: diabetic nephropathy
ACCORD: Action to Control Cardiovascular Risk in Diabetes
IDCD: Israel Diabetes and Cognitive Decline
AD: Alzheimer disease
rtCGM: real-time continuous glucose monitoring
GLP-1 RA: glucagon-like peptide 1 receptor agonist
DPP4: dipeptidyl-peptidase 4
SGLT2: sodium glucose cotransporter 2
